# Biofilm Formation, Virulence Traits, and Antimicrobial Resistance Profiles of *Enterococcus faecalis* in Layer Parent Stock in Bangladesh

**DOI:** 10.1155/ijm/4082070

**Published:** 2026-01-08

**Authors:** Nirab Chakroborty, Naeem Ahammed Ibrahim Fahim, Md Saiful Islam, Md. Liton Rana, Farhana Binte Ferdous, Md. Nowshad Atiq, Md. Abdus Sobur, Mahfuz Ahammed, Sukumar Saha, Md. Tanvir Rahman

**Affiliations:** ^1^ Department of Microbiology and Hygiene, Faculty of Veterinary Science, Bangladesh Agricultural University, Mymensingh, Bangladesh, bau.edu.bd; ^2^ Department of Animal Science, University of California–Davis, Davis, California, USA; ^3^ University of Chinese Academy of Sciences, Beijing, China, ucas.ac.cn; ^4^ National Engineering Research Center of Industrial Wastewater Detoxication and Resource Recovery, Research Center for Eco-Environmental Sciences, Chinese Academy of Sciences, Beijing, China, cas.cn

**Keywords:** antimicrobial resistance, Bangladesh, biofilm formation, *Enterococcus faecalis*, layer parent stock, multidrug resistance, virulence genes

## Abstract

*Enterococcus faecalis* is an opportunistic pathogen of growing concern in both human and veterinary medicine due to its virulence traits, biofilm‐forming ability, and resistance to multiple antibiotics. This study was aimed at investigating the occurrence, virulence factors, biofilm formation, and antimicrobial resistance (AMR) of *E. faecalis* in layer parent stock birds in Bangladesh. Samples (*n* = 80) were collected from healthy (cloacal swabs, *n* = 60) and dead (liver tissues, *n* = 20) birds. PCR was used for *E. faecalis* confirmation and detection of virulence genes. Biofilm formation was assessed using Congo red agar, and antimicrobial susceptibility was determined by disc diffusion. *E. faecalis* was detected in 76.3% of samples, with higher detection in live birds (80%) than in dead birds (65%). Biofilm production was found in 75.4% of isolates, with a higher rate in dead birds (84.6%) than live birds (72.9%). Strong and intermediate biofilm‐forming capacities were more prevalent in isolates from dead birds. All eight tested virulence genes were commonly distributed, particularly *pil* (95.8%), *ace* (93.4%), and *agg* (91.8%), with no significant differences between live and dead bird isolates. High resistance was observed against ampicillin (93.4%), ciprofloxacin (80.3%), erythromycin (78.7%), and tetracycline (72.1%). Multidrug resistance (MDR) was found in 79.2% of isolates from live birds and 69.2% from dead birds, with multiple antibiotic resistance indices ranging from 0.27 to 0.72. To the best of our knowledge, this is the first study in Bangladesh determining MDR and virulence determinants in *E. faecalis* isolates from layer parent stock. These findings highlight *E. faecalis* as a prevalent, multidrug‐resistant, and virulent bacterium in breeder flocks, emphasizing the need for routine AMR monitoring in parent stock farms.

## 1. Introduction

*Enterococcus faecalis* is a Gram‐positive, facultative anaerobic bacterium that constitutes a natural component of the gastrointestinal flora of humans and animals [1]. Although typically commensal, *E. faecalis* can become opportunistic, causing a broad spectrum of infections, including urinary tract infections, endocarditis, intra‐abdominal infections, and bacteremia, particularly in healthcare settings [2, 3]. It is ranked among the top three pathogens associated with nosocomial infections [4]. Vancomycin‐resistant enterococci (VRE), comprising *E. faecalis* and *E. faecium*, are major contributors to antimicrobial resistance (AMR) in hospital settings, with *E. faecium* more frequently implicated in outbreaks [5]. *E. faecalis* is a significant cause of infective endocarditis, especially in elderly and immunocompromised individuals, contributing to increased mortality [6]. Beyond healthcare settings, *E. faecalis* has been detected in food‐producing animals, particularly poultry, raising concerns over zoonotic transmission [7]. Its role in urinary tract infections and multidrug resistance has made it a critical pathogen globally, with isolates from food animals increasingly exhibiting virulence and resistance profiles similar to human clinical strains [8, 9].

One of the key survival mechanisms of *E. faecalis* is its ability to form biofilms, which significantly enhances its resistance to host immune defenses and a wide range of antimicrobials, thereby enabling persistence in both host tissues and environmental niches [10]. Biofilm formation facilitates chronic infections and increases resistance to disinfection and antibiotic treatment, particularly in hospital settings [10, 11]. The regulation of biofilm formation is complex and involves quorum‐sensing systems such as the *fsr* operon, which modulates the expression of virulence genes like *gelE* and *sprE* [12]. Additionally, several adhesins and surface proteins, encoded by genes such as *agg*, *ace*, and *pil*, promote adherence to host tissues and abiotic surfaces, enhancing colonization potential [13, 14]. These virulence traits, often located on plasmids, transposons, or pathogenicity islands, can be horizontally transferred across strains or species, accelerating the dissemination of pathogenic determinants [15, 16]. This genetic plasticity, coupled with biofilm‐mediated persistence, contributes to the emergence of multidrug‐resistant (MDR) and highly virulent *E. faecalis* clones in both clinical and nonclinical environments.

The rise of AMR in *E. faecalis* presents a growing threat to public and animal health, complicating treatment options and limiting the efficacy of first‐line antibiotics. *E. faecalis* strains frequently exhibit resistance to aminoglycosides, tetracyclines, macrolides, and *β*‐lactams, with emerging reports of reduced susceptibility to vancomycin and linezolid, which are critical drugs for treating resistant Gram‐positive bacterial infections [17, 18]. The misuse and overuse of antibiotics in both human medicine and animal agriculture are key drivers of resistance selection and propagation [19]. In livestock environments, resistant strains can persist in soil, water, and fecal matter, facilitating indirect transmission to humans via food, occupational exposure, or environmental contamination [20]. Moreover, surveillance studies have revealed similarities between *E. faecalis* isolates from food animals and human clinical strains in terms of resistance genes and virulence profiles, indicating the possibility of interspecies transmission [7, 21].

Bangladesh, characterized by high population density and rapidly expanding poultry production, presents an ideal setting for the dissemination of antimicrobial‐resistant bacteria from animals to humans. Layer parent stock, a foundational element in poultry breeding, serves as a potential reservoir for enteric pathogens, including *E. faecalis* [22]. These breeder flocks play a pivotal role in the vertical transmission of microbes to commercial layers and broilers, heightening food safety concerns [23]. Although *E. faecalis* has been reported in fish, wild animals, and cattle in Bangladesh [24–26], its characterization in the layer parent stock remains uninvestigated. Given the potential for transmission through the poultry production chain, it is critical to assess the resistance traits and virulence potential of *E. faecalis* from breeder flocks. Therefore, the present study was aimed at isolating and characterizing *E. faecalis* from layer parent stock in Bangladesh, focusing on their AMR profiles, virulence gene determination, and biofilm‐forming ability.

## 2. Materials and Methods

### 2.1. Ethical Approval

The Animal Welfare and Experimentation Ethics Committee at Bangladesh Agricultural University, Mymensingh, approved the methods described in this work [Approval Number AWEEC/BAU/2024(2)/20(a)].

### 2.2. Sample Collection and Processing

**Figure 1:**
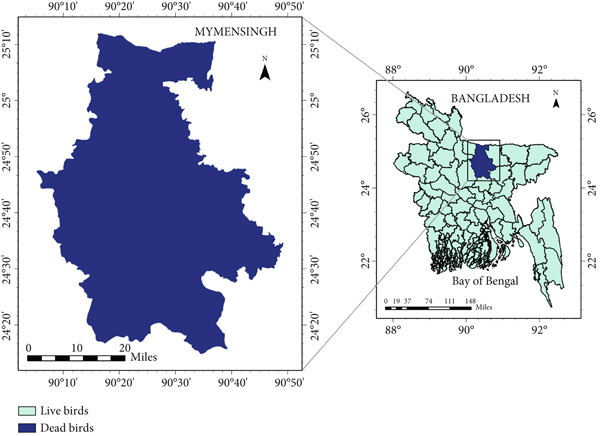
Map showing the sampling areas of this study in Mymensingh District, Bangladesh.

This study was carried out in Bhaluka Upazila, Mymensingh District, Bangladesh (24.3789°N, 90.19373°E), a region with a high density of commercial layer breeder farms (Figure 1). Sample collection was conducted over 4 months, from October 2023 to January 2024, coinciding with the late autumn to winter season when poultry flocks are more vulnerable to stress‐related infections.

All samples were obtained from a single commercial layer parent stock farm composed of three sheds, each housing around 3000 birds aged between 26 and 34 weeks. Information on recent antimicrobial treatments was not available, as the farm did not provide antibiotic use records for the flock at the time of sampling. Sampling involved both live and dead birds, totaling 80 individuals. Recently dead birds were included to compare *E. faecalis* from clinically affected birds with those from healthy birds. Although the exact causes of death were not confirmed, postmortem examinations showed lesions such as air sacculitis and fibrinous deposits on the liver, suggesting bacterial involvement. These carcasses were therefore selected for bacterial isolation and characterization. Cloacal swabs were collected from 60 live birds that appeared clinically healthy, showing no visible signs of disease at the time of collection. Sampling was performed under aseptic conditions by a team comprising two licensed veterinarians and one microbiologist. Twenty carcasses of birds that had recently died on the farm were also included in the study. Carcasses of the dead birds were immediately transported to the Department of Microbiology and Hygiene (DMH), Faculty of Veterinary Science, Bangladesh Agricultural University, for postmortem examination. To ensure biosample integrity, both cloacal swabs and carcasses were placed in separate sterile containers and transported using insulated iceboxes equipped with frozen gel packs. The internal temperature of the transport containers was maintained between 4°C and 8°C using a validated cold chain protocol. All samples were delivered to the laboratory within 2–3 h of collection.

Upon arrival at the laboratory, postmortem examinations were carried out by two veterinarians. Birds exhibiting gross pathological lesions, such as air sacculitis and fibrinous membranes on the liver surface, were selected for further investigation. Swabs from the affected hepatic tissue were aseptically collected and immediately placed into sterile tubes containing 5 mL of sterile nutrient broth (HiMedia, Mumbai, Maharashtra, India). All swab samples were thoroughly vortexed and incubated overnight at 37°C in nutrient broth to facilitate bacterial enrichment prior to the isolation of *E. faecalis*.

### 2.3. Isolation and Molecular Detection of *E. faecalis*

Following overnight enrichment, 10 *μ*L of each culture was inoculated onto *Enterococcus* agar base (HiMedia, Mumbai, Maharashtra, India) and incubated aerobically at 37°C for 24 h. A single pure colony displaying typical *E. faecalis* morphology, characterized by distinct, tiny, oval‐shaped yellowish colonies, was selected and subjected to Gram staining and preliminary biochemical screening. Isolates that were catalase‐negative and positive in the pyrrolidonyl aminopeptidase (PYR) test were considered presumptive *E. faecalis* [27]. These isolates were preserved in 50% glycerol at −20°C for subsequent analysis.

Genomic DNA was extracted using a simplified heat lysis protocol [28, 29]. Briefly, 1 mL of the enriched broth was centrifuged at 5000 rpm for 5 min. The pellet was resuspended in 200 *μ*L of sterile phosphate‐buffered saline (PBS) and mixed thoroughly. The suspension was then boiled for 10 min, cooled for another 10 min, and centrifuged at 10,000 rpm for 10 min. The supernatant, containing crude genomic DNA, was collected and stored at −20°C.

Confirmation of *E. faecalis* was carried out by PCR amplification targeting the *ddl* gene, using species‐specific primers (Table 1). Briefly, PCR tests were performed using a 20‐*μ*L PCR mixture that contained 5 *μ*L of DNA template, 10 *μ*L of master mix (Promega, Madison, Wisconsin, United States), 3 *μ*L of nuclease‐free water, and 1 *μ*L of forward and reverse primers (Table 1). A UV transilluminator (Biometra, Göttingen, Germany) was used to view the PCR results after resolution on a 1.5% agarose gel and ethidium bromide staining. To confirm the anticipated amplicon sizes, a 100‐bp DNA ladder (Promega, Madison, Wisconsin, United States) was used as a size marker. The thermal profile for PCR of the *ddl* gene was as follows: initial denaturation at 94°C for 5 min, 30 cycles of denaturation at 95°C for 30 s, annealing at 54°C for 30 s, and extension at 72°C for 1 min and then the final extension at 72°C for 5 min, with a holding temperature of 4°C.

**Table 1:** Primers used in this study to identify *Enterococcus faecalis* and their virulence genes.

**Targeted factors**	**Targeted genes**	**Primer sequences (5** ^′^ **-3** ^′^ **)**	**Annealing Tm (°C)**	**Size (bp)**	**References**
*E. faecalis*	*ddl_E. faecalis_*	F: ATCAAGTACAGTTAGTCTT	50	941	[30]
		R: ACGATTCAAAGCTAACTG			

Virulence	*agg*	F: TCTTGGACACGACCCATGAT	58	413	[31]
		R: AGAAAGAACATCACCACGAC			
	*fsrA*	F: CGTTCCGTCTCTCATAGTTA	53	474	
		R: GC*AGG*ATTTG*AGG*TTGCTAA			
	*fsrB*	F: TAATCT*AGG*CTTAGTTCCCAC	55		
		R: CTAAATGGCTCTGTCGTCTAG			
	*gelE*	F: GGTGAAGAAGTTACTCTGAC	52	704	
		R: GGTATTGAGTTATG*AGG*GGC			
	*ace*	F: GAATGACCGAGAACGATGGC	58	615	
		R: CTTGATGTTGGCCTGCTTCC			
	*pil*	F: GAAGAAACCAAAGCACCTAC	53	620	
		R: CTACCTAAGAAAAGAAACGG			
	*fsrC*	F: GTGTTTTTGATTTCGCCAGAGA	54	716	
		R: TATAACAATCCCCAACCGTG			
	*sprE*	F: CTGAGGACAGAAGACAAGAG	53	432	
		R: GGTTTTTCTCACCTGGATAG			

### 2.4. Assessment of Biofilm Formation by *E. faecalis*

The ability of *E. faecalis* isolates to produce biofilms was evaluated phenotypically using the Congo red agar (CRA) method, as described in previous studies [32, 33]. Briefly, isolates were streaked onto CRA plates and incubated at 37°C for 24 h. Colony morphology was then used to classify biofilm production capacity. Isolates forming dry, black, and rough colonies were categorized as strong biofilm producers. Those producing colonies with a pink background and dark centers were considered moderate producers, while smooth, pink colonies indicated a lack of biofilm formation [33, 34]. For the assessment of biofilm formation using the CRA method, each plate was independently evaluated by three observers to reduce subjective bias, and the experiment was repeated twice to confirm the consistency of results.

### 2.5. Molecular Detection of Virulence‐Associated Genes in *E. faecalis*

The detection of selected virulence genes commonly found in *E. faecalis*, including *fsrA*, *fsrB*, *fsrC*, *pil*, *gelE*, *sprE*, *ace*, *agg*, and *cyl*, was performed using simplex PCR assays (Table 1). PCR amplification conditions were consistent with those used for species identification, as described in Section 2.3. Previously confirmed *E. faecalis* strains harboring the respective virulence genes served as positive controls. Sterile deionized water was used as the template in no‐template reactions to serve as negative controls.

### 2.6. Antimicrobial Susceptibility Testing of *E. faecalis*

Antibiotic susceptibility of *E. faecalis* isolates was evaluated using the disc diffusion method [35], following Clinical and Laboratory Standards Institute (CLSI) guidelines [36]. For this assay, colonies grown on *Enterococcus* agar base were incubated at 37°C for 18–24 h. A few (2–3) well‐isolated colonies were suspended in 0.85% sterile normal saline and adjusted to match a 0.5 McFarland turbidity standard. The standardized bacterial suspension was evenly spread onto Mueller–Hinton agar plates using sterile cotton swabs. Antibiotic discs were then placed on the surface, and the plates were incubated at 37°C for another 24 h.

A total of 11 antibiotic discs (HiMedia, Mumbai, Maharashtra, India), representing nine antibiotic classes, were used in this study. The selection included antibiotics from the WHO′s Access, Watch, and Reserve (AWaRe) classification:

• Access group: ampicillin (10 *μ*g), tetracycline (30 *μ*g), and chloramphenicol (30 *μ*g)
• Watch group: vancomycin (30 *μ*g), erythromycin (15 *μ*g), ciprofloxacin (5 *μ*g), levofloxacin (5 *μ*g), norfloxacin (10 *μ*g), and rifampin (5 *μ*g)
• Reserve group: fosfomycin (200 *μ*g) and linezolid (30 *μ*g)

*Escherichia coli* ATCC 25922 was used as the quality control strain to ensure the reliability of the test. Isolates exhibiting resistance to at least one agent in three or more antimicrobial categories were designated as MDR [37].

The multiple antibiotic resistance (MAR) index for each isolate was determined by dividing the number of antibiotics to which the isolate exhibited resistance by the total number of antibiotics tested [38]. This index provides an estimate of the exposure risk of bacteria to antibiotics in the environment from which the isolates were obtained [38].

### 2.7. Data Processing and Statistical Evaluation

Data from this study were organized using Microsoft Excel 365 (Microsoft, Redmond, Washington, United States) and analyzed in R software (Version 4.3.0). Descriptive statistics were used to calculate the prevalence of *E. faecalis* isolates and associated characteristics. Binomial 95% confidence intervals (CI_95_) for prevalence estimates were computed using base R functions.

Group comparisons, such as differences in isolation rates across sample types, variation in biofilm production levels, and associations between biofilm formation and the presence of virulence genes or antibiotic resistance, were assessed using the chi‐square test. When significant differences were detected, Tukey′s post hoc test was applied to identify specific group‐level differences.

Bivariate analyses were also performed to explore potential associations between individual virulence genes and between resistance to different antibiotic agents. A *p* value of less than 0.05 was considered statistically significant throughout the analysis.

## 3. Result

### 3.1. Occurrence of *E. faecalis*

In PCR analysis, *E. faecalis* was detected in 76.3% (61/80; CI_95_: 65.2, 84.8) of the samples, where the detection rate of the isolates was higher (*p* > 0.05) in live birds (80%; CI_95_: 67.3, 88.8) than in dead birds (65%; CI_95_: 40.9, 83.7).

### 3.2. Determination of Biofilm‐Forming Abilities in the Isolated *E. faecalis*

**Figure 2:**
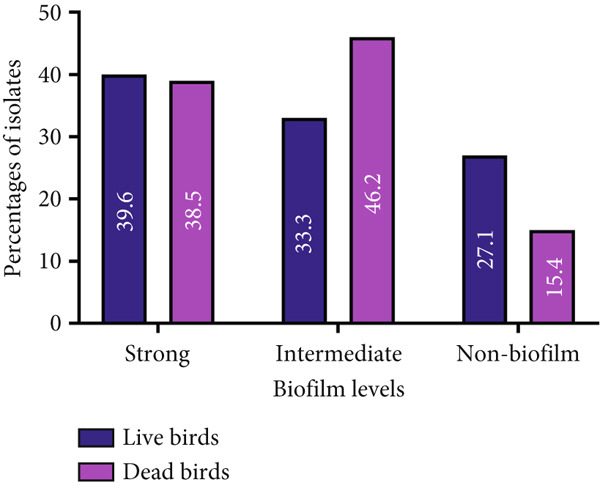
Biofilm‐forming capacity of *Enterococcus faecalis* isolates from live and dead parent stock birds.

In the CRA plate test, 75% (46/61; CI_95_: 62.4, 85.2) of the *E. faecalis* isolates were biofilm formers, where isolates from dead birds (84.6%; CI_95_: 53.7, 97.3) showed higher (*p* > 0.05) biofilm‐forming capabilities than isolates from live birds (72.9%; CI_95_: 57.9, 84.3). Moreover, strong biofilm‐forming ability was observed in 39.6% (19/48; CI_95_: 26.1, 54.7) of the 48 isolates from live birds, while 33.3% (16/48; CI_95_: 20.8, 48.5) showed intermediate biofilm formation. The remaining 27.1% (13/48; CI_95_: 15.7, 42.1) were classified as nonbiofilm producers. Similarly, out of 13 PCR‐positive *E. faecalis* isolates from dead birds, 38.5% (5/13; CI_95_: 15.1, 67.7) demonstrated strong biofilm formation, 46.2% (6/13; CI_95_: 20.4, 73.9) exhibited intermediate biofilm formation, and 15.4% (2/13; CI_95_: 2.7, 46.3) were identified as nonbiofilm producers (Figure 2).

### 3.3. Profiles of Virulence Genes in *E. faecalis*

Among the 61 *E. faecalis* isolates, the most frequently detected virulence genes were *pil* (95.8%: 95.8% in live vs. 92.3% in dead birds), *ace* (93.4%: 93.8% vs. 92.3%), *agg* (91.8%: 91.7% vs. 92.3%), and *fsrC* (91.8%: 91.7% vs. 92.3%). Other detected genes included *fsrA* (83.6%: 83.3% vs. 84.6%), *gelE* (83.6%: 83.3% vs. 84.6%), *sprE* (82.0%: 81.3% vs. 84.6%), and *fsrB* (78.7%: 79.2% vs. 76.9%). No statistically significant differences (*p* > 0.05) were observed between isolates from live and dead birds for any of the virulence genes (Table 2).

**Table 2:** Occurrence of virulence genes in *Enterococcus faecalis* isolates from healthy and dead parent stock birds.

**Virulence genes**	**Total no. of positive isolates (%) [95% CI]**		**p** **value**	**Total no. of positive isolates (%) [95% CI] (** **N** = 61**)**
	**Live (cloacal swab) (** **N** = 48**)**	**Dead (liver swabs) (** **N** = 13**)**		
*agg*	44 (91.7) [79.1, 97.3]	12 (92.3) [62.1, 99.6]	> 0.05	56 (91.8) [81.2, 96.9]
*ace*	45 (93.8) [81.8, 98.4]	12 (92.3) [62.1, 99.6]	> 0.05	57 (93.4) [83.3, 97.9]
*fsrA*	40 (83.3) [69.2, 92.0]	11 (84.6) [53.7, 97.3]	> 0.05	51 (83.6) [71.5, 91.4]
*fsrB*	38 (79.2) [64.6, 89.0]	10 (76.9) [45.9, 93.8]	> 0.05	48 (78.7) [65.9, 87.7]
*fsrC*	44 (91.7) [79.1, 97.3]	12 (92.3) [62.1, 99.6]	> 0.05	56 (91.8) [81.2, 96.9]
*gelE*	40 (83.3) [69.2, 92.0]	11 (84.6) [3.7, 97.3]	> 0.05	51 (83.6) [71.5, 91.4]
*sprE*	39 (81.3) [66.9, 90.6]	11 (84.6) [3.7, 97.3]	> 0.05	50 (81.9) [69.6, 90.2]
*pil*	46 (95.8) [84.6, 99.3]	12 (92.3) [62.1, 99.6]	> 0.05	58 (95.8) [85.4, 98.7]

*Note:* A *p* value of less than 0.05 was considered statistically significant.

Abbreviation: CI, confidence interval.

In the bivariate analysis, the results from live birds indicated a statistically significant strong correlation between the *gelE* and *fsrA* genes (Spearman correlation coefficient, *ρ* = 1; *p* < 0.01). Additionally, a correlation was observed between the *sprE* and *ace* genes (*ρ* = 0.3; *p* < 0.05), while in dead birds, the study′s findings revealed a statistically significant association between the *fsrA* and *agg* genes (*ρ* = 0.7; *p* < 0.05). Furthermore, there was a significant association between the *fsrB* and *agg* genes (*ρ* = 0.5; *p* < 0.05).

### 3.4. AMR Profiles of *E. faecalis* Isolates From Live and Dead Birds

Among the 61 *E. faecalis* isolates, the highest phenotypic resistance was observed to ampicillin (93.4%; 93.8% in live vs. 92.3% in dead birds), followed by erythromycin (78.7%; 85.4% vs. 53.9%) and ciprofloxacin (80.3%; 85.4% vs. 61.5%). Resistance to tetracycline was also common (72.1%; 75.0% vs. 61.5%). Notable resistance was recorded for norfloxacin (26.2%; 25.0% vs. 30.8%), rifampin (36.1%; 37.5% vs. 30.8%), and levofloxacin (19.7%; 18.8% vs. 23.1%). Lower resistance rates were observed for chloramphenicol (8.2%), vancomycin (8.2%), fosfomycin (6.6%), and linezolid (9.8%). A statistically significant difference (*p* = 0.014) was found in erythromycin resistance between live and dead bird isolates, while differences in other antimicrobials were not significant (*p* > 0.05) (Table 3).

**Table 3:** Antimicrobial resistance patterns in *Enterococcus faecalis* isolated from live and dead parent stock birds.

**Antimicrobial group**	**Antimicrobial class**	**Antimicrobial agents**	**Total no. of positive isolates (%) [95% CI]**		**p** **value**	**Total no. of positive isolates (%) [95% CI] (** **N** = 61**)**
			**Live birds (** **N** = 48**)**	**Dead birds (** **N** = 13**)**		
Access	Penicillins	Ampicillin	45 (93.8) [81.8, 98.4]	12 (92.3) [62.1, 99.6]	> 0.05	57 (93.4) [83.3, 97.9]
	Amphenicols	Chloramphenicol	4 (8.3) [2.7, 20.9]	1 (7.7) [0.4, 37.9]	> 0.05	5 (8.2) [3.1, 18.8]
	Tetracyclines	Tetracycline	36 (75) [60.1, 85.9]	8 (61.5) [32.3, 84.9]	> 0.05	44 (72.1) [58.9, 82.5]

Watch	Glycopeptides	Vancomycin	4 (8.3) [2.7, 20.9]	1 (7.7) [0.4, 37.9]	> 0.05	5 (8.2) [3.1, 18.8]
	Macrolides	Erythromycin	41 (85.4) [71.6, 93.5]	7 (53.9) [26.1, 79.6]	< 0.05	48 (78.7) [65.9, 87.7]
	Fluoroquinolones	Ciprofloxacin	41 (85.4) [71.6, 93.5]	8 (61.5) [32.3, 84.9]	> 0.05	49 (80.3) [67.8, 88.9]
		Levofloxacin	9 (18.8) [9.4, 33.1]	3 (23.1) [6.2, 54.0]	> 0.05	12 (19.7) [11.0, 32.2]
		Norfloxacin	12 (25) [14.1, 39.9]	4 (30.8) [10.4, 61.1]	> 0.05	16 (26.2) [16.2, 39.3]
	Fosfomycins	Fosfomycin	3 (6.3) [1.6, 18.2]	1 (7.7) [0.4, 37.9]	> 0.05	4 (6.6) [2.1, 16.7]
	Ansamycins	Rifampin	18 (37.5) [24.3, 52.7]	4 (30.8) [10.4, 61.1]	> 0.05	22 (36.1) [24.5, 49.4]

Reserve	Oxazolidinones	Linezolid	3 (6.3) [1.6, 18.2]	3 (23.1) [6.2, 54.0]	> 0.05	6 (9.8) [4.1, 20.8]

*Note:* Values with different superscripts differ significantly (*p* < 0.05) within the variable under assessment.

Abbreviation: CI, confidence interval.

In live birds, the bivariate analysis demonstrated that a high positive significant correlation existed between resistance patterns of erythromycin and tetracycline (*ρ* = 0.4; *p* < 0.01), ciprofloxacin and tetracycline (*ρ* = 0.3; *p* < 0.01), ciprofloxacin and levofloxacin (*ρ* = 0.6; *p* < 0.01), norfloxacin and ciprofloxacin (*ρ* = 0.7; *p* < 0.01), norfloxacin and levofloxacin (*ρ* = 0.8; *p* < 0.01), and tetracycline and norfloxacin (*ρ* = 0.3; *p* < 0.05). However, in dead birds, a strong correlation was found between norfloxacin and levofloxacin (*ρ* = 0.8; *p* < 0.01).

Nineteen types of multidrug resistance patterns of *E. faecalis* isolates from healthy birds with AMP‐E‐TE and AMP, E, TE, CIP, LEVA, and NOR were the most common patterns. Of 48 PCR‐positive isolates, 79.17% (38/48) showed multidrug resistance, while 69.23% of isolates from dead birds showed multidrug resistance patterns. The MAR value varied from 0.27 to 0.72 for healthy and dead bird isolates (Table 4).

**Table 4:** Multidrug resistance pattern and multiple antibiotic resistance index value of *E. faecalis* isolates from live and dead parent stock birds in Bangladesh.

**Sample type**	**Antibiotic resistance pattern**	**No. of antibiotics (classes)**	**No. of isolates**	**Overall, MDR isolates %**	**MAR index**
Healthy birds	AMP, E, RA	3 (3)	3	38/48 (79.17%)	0.27
	AMP, E, TE, CIP, RA	5 (5)	1		0.45
	AMP, E, TE, C, LNZ	5 (5)	1		0.45
	AMP, E, TE, CIP, LEVA, NOR, C	7 (5)	1		0.64
	AMP, E, TE	3 (3)	7		0.27
	AMP, E, TE, CIP, LEVA, NOR, C	7 (5)	1		0.64
	AMP, E, TE, CIP, LEVA, NOR	6 (4)	7		0.55
	AMP, VA, CIP, RA	4 (4)	1		0.36
	AMP, TE, CIP, LEVA, NOR, RA	6 (4)	1		0.55
	AMP, E, TE, CIP	4 (4)	3		0.36
	AMP, E, TE, RA	4 (4)	4		0.36
	AMP, VA, E, TE, RA	5 (5)	1		0.45
	AMP, E, TE, CIP, LEVA, NOR, RA, LNZ	8 (6)	1		0.72
	E, TE, RA, LNZ	4 (4)	1		0.36
	AMP, E, TE, CIP, NOR	5 (4)	1		0.45
	AMP, E, TE, CIP, NOR, FOS, C	7 (6)	2		0.64
	AMP, VA, E, TE, FOS	5 (5)	1		0.45
	E, TE, FOS	3 (3)	1		0.27

Dead birds	AMP, E, RA	3 (3)	1	9/nn (69.23)	0.27
	E, TE, CIP, NOR, LNZ	5 (4)	1		0.45
	AMP, CIP, RA	3 (3)	1		0.27
	AMP, E, TE, CIP, LEV, NOR, RA, LNZ	8 (6)	1		0.72
	AMP, TE, CIP, LNZ	4 (4)	1		0.36
	AMP, TE, CIP, LEV, NOR, RA, FOS	7 (5)	1		0.64
	AMP, E, TE, CIP, LEV, NOR	6 (4)	1		0.55
	AMP, E, TE	3 (3)	2		0.27

Abbreviations: MAR, multiple antibiotic resistance; MDR, multidrug resistance.

## 4. Discussion

The current study provides a detailed characterization of *E. faecalis* isolates from layer parent stock birds, focusing on their prevalence, biofilm formation, virulence gene distribution, and AMR profiles. To the best of our knowledge, this is the first study in Bangladesh focusing on the detection of *E. faecalis* in layer parent stock and their characterization. It is important to note that the comparisons between isolates from live (cloacal) and dead (hepatic) birds were intended to be descriptive rather than inferential. Our goal was not to distinguish between colonization and infection, nor to attribute mortality to *E. faecalis*. Instead, including both sample types allowed us to characterize the diversity, virulence traits, and AMR patterns of *E. faecalis* circulating within the same parent stock flock. Therefore, the differences observed between sample types should be interpreted as exploratory observations rather than indicators of pathogenic causality.

The overall detection rate of *E. faecalis* was high (76.3%) across all samples, with a slightly greater occurrence in live birds compared to dead birds. However, this difference was not statistically significant. Previously, Mudenda et al. [39] performed a similar study in poultry, reporting *Enterococcus* species in 84.4% of the samples. However, Mwikuma et al. [40] and Velhner et al. [41] reported lower detection rates of *E. faecalis* in poultry samples compared to our findings, with prevalence rates of 37.9% and 37.5%, respectively. The higher detection rate of *E. faecalis* in our study compared to those two studies may be attributed to differences in bird types, sample sources, diagnostic sensitivity (PCR targeting the *ddl* gene with prior enrichment), environmental conditions, and antimicrobial use practices; additionally, parent stock birds may harbor higher bacterial loads due to longer lifespan and increased environmental exposure. The relatively high carriage rate among live birds is of particular importance, as these birds are typically asymptomatic yet may serve as ongoing sources of environmental contamination and vertical or horizontal transmission [42]. The lower detection rate in dead birds could be attributed to the presence of competing pathogens in terminal infections or possible postmortem microbial changes affecting PCR sensitivity [43]. Nevertheless, the consistent presence of *E. faecalis* in both groups highlights the bacterium′s ecological adaptability and potential for persistent colonization in poultry populations.

Biofilm formation is a well‐recognized survival and virulence strategy of *E. faecalis*, contributing to its ability to persist in hostile environments, evade host immune responses, and resist antimicrobial therapy [33]. In the present study, 75.4% of *E. faecalis* isolates exhibited biofilm‐forming capacity, as determined by the CRA assay. Although the difference was not statistically significant, a higher proportion of isolates from dead birds (84.6%) were biofilm producers compared to those from live birds (72.9%), suggesting a potential association between biofilm formation and disease severity [44]. Further classification by biofilm‐forming intensity revealed that isolates from dead birds had a slightly higher frequency of strong and intermediate biofilm formation than those from live birds. This pattern may reflect the enhanced adaptability of pathogenic strains under stress conditions within the host, especially during systemic infections [45]. Biofilm formation facilitates adhesion to host tissues and surfaces such as the intestinal mucosa or internal organs, while simultaneously protecting against phagocytosis, oxidative stress, and antibiotic penetration. This makes infections caused by biofilm‐producing strains more persistent and challenging to treat, especially when standard therapies fail to reach adequate concentrations within the biofilm matrix [46]. Notably, the presence of biofilm‐forming *E. faecalis* in healthy birds implies that colonized individuals may serve as long‐term carriers and silent reservoirs, shedding bacteria into the environment or transmitting them through direct contact, fomites, or vertical transmission routes [47]. Such carriers pose a challenge for disease control at the farm level, especially in parent stock populations that contribute to the downstream poultry production system.

The widespread detection of multiple virulence genes across *E. faecalis* isolates from both live and dead birds suggests that these strains carry a substantial pathogenic potential, regardless of the host′s clinical status. The high prevalence of genes related to adhesion (*agg*, *ace*, and *pil*) and quorum‐sensing regulation (*fsrA*, *fsrB*, and *fsrC*) reflects the species′ intrinsic ability to colonize host tissues, coordinate biofilm formation, and persist in the avian host environment [31]. Importantly, the consistent presence of these genes in isolates from both healthy and diseased birds highlights the risk of silent carriage in apparently healthy parent stock, which may contribute to horizontal transmission within flocks. The absence of statistically significant differences in gene distribution between groups indicates that virulence gene carriage is likely stable and not solely associated with overt disease. Still, it may act as a predisposing factor under stress or immunosuppression. The observed correlations between key genes, such as between *gelE* and *fsrA* and between *agg* and the *fsr* operon components, suggest coordinated regulatory mechanisms that may enhance colonization and persistence. These interactions support the notion that pathogenicity in *E. faecalis* is multifactorial, involving tightly linked adhesion, regulation, and enzymatic activity [48].

The antimicrobial susceptibility patterns observed among *E. faecalis* isolates in this study raise significant public health concerns. High resistance rates to several commonly used antibiotics, including ampicillin, erythromycin, ciprofloxacin, and tetracycline, highlight the persistent selection pressure likely exerted by widespread antimicrobial use in poultry production. Moreover, the high resistance to multiple first‐line drugs not only limits treatment options but also reflects the resilience and adaptability of *E. faecalis* in farm environments. Although most resistance rates did not significantly differ between live and dead bird isolates, the notably higher erythromycin resistance in live bird isolates may indicate ongoing selective exposure in subclinical carriers. This is especially troubling, as live birds with high resistance levels may silently disseminate resistant strains across the flock and beyond. These findings are consistent with earlier studies conducted across various regions. Previous reports have similarly demonstrated that the majority of *E. faecalis* isolates exhibit resistance to one or more antibiotics, including those from poultry samples in South Korea and Zambia [9, 40]. In particular, increased resistance to tetracycline, erythromycin, and ciprofloxacin has been reported among Enterococci in Zambian poultry flocks [39], while resistance to tetracycline, penicillin, and ciprofloxacin was also observed in the same region [40]. High resistance to tetracycline has been a recurring observation in multiple studies [9, 49], reinforcing its role as a heavily impacted drug class. Additionally, our erythromycin resistance levels are comparable to those reported by Fracalanzza et al. [50], who documented erythromycin resistance in 82% of enterococcal isolates.

Correlations among resistance phenotypes, particularly among fluoroquinolones and tetracyclines, point to potential coselection or coresistance mechanisms. The concurrent resistance patterns suggest the presence of linked genetic elements, such as plasmids or transposons, facilitating the spread of resistance genes across classes [51]. These associations raise concerns about the long‐term efficacy of antimicrobials that are critical for both veterinary and human medicine. The high proportion of MDR isolates and elevated MAR indices further underscores the risk posed by these strains. The high prevalence of multidrug resistance, observed in 79.17% of isolates from live birds and 69.23% from dead birds, along with MAR index values ranging from 0.27 to 0.72, points toward prolonged and repeated antibiotic exposure in the farm environment. Previously, [40] showed that 67% of *E. faecalis* isolates were MDR in nature. The presence of MDR *E. faecalis* isolates with higher MAR indices in both healthy and diseased birds, especially in a parent stock setting, has significant implications for vertical transmission and farm‐level persistence. These findings highlight the urgent need for prudent antimicrobial use, enhanced biosecurity, and ongoing surveillance in poultry systems to mitigate the development and spread of resistant *E. faecalis*.

In the dead birds, postmortem examinations revealed lesions such as air sacculitis and fibrinous material on the liver surface, which are generally indicative of bacterial involvement. Although we did not confirm the cause of death or perform diagnostics to directly link these findings to *E. faecalis*, the presence of biofilm‐forming and antimicrobial‐resistant isolates in these birds is noteworthy. Biofilm‐producing enterococci are known to persist in host tissues and may contribute to chronic or difficult‐to‐resolve infections when conditions are favorable [52]. Similarly, AMR can complicate clearance of opportunistic bacteria, potentially increase the risk of secondary infections, or contribute to disease severity under stress or immunosuppression [53]. While our data do not establish a causal relationship, these observations highlight the importance of monitoring resistant and virulent enterococci in breeder flocks, where compromised birds may be more susceptible to the consequences of opportunistic pathogens.

This study faced several limitations due to funding constraints. Quantitative biofilm assessment methods such as crystal violet staining or spectrophotometric assays were not used, limiting the precision of biofilm characterization. Minimum inhibitory concentration (MIC) testing was also not performed, which restricted detailed analysis of AMR levels. Additionally, genotypic profiling of resistance genes was not included, preventing a deeper understanding of the molecular basis of resistance. Incorporating advanced techniques like whole‐genome sequencing in future studies would provide a more comprehensive view of the resistance mechanisms and virulence potential of *E. faecalis*.

## 5. Conclusions

This study highlights the widespread presence of *E. faecalis* in layer parent stock birds, with a high proportion of isolates exhibiting virulence gene carriage, biofilm‐forming ability, and multidrug resistance. The detection of multiple virulence genes in both healthy and dead birds suggests that these isolates possess intrinsic pathogenic potential, regardless of clinical signs. Biofilm formation, particularly in isolates from dead birds, may contribute to disease severity and persistence within poultry environments. The high resistance to commonly used antimicrobials, combined with strong correlations among resistance traits and frequent multidrug resistance patterns, underscores the threat posed by these strains. These findings emphasize the need for enhanced antimicrobial stewardship, routine surveillance, and the implementation of integrated biosecurity measures in poultry production systems. Future research incorporating genotypic resistance profiling and broader epidemiological coverage will be essential to better understand the transmission dynamics and public health implications of *E. faecalis* in poultry.

## Conflicts of Interest

The authors declare no conflicts of interest.

## Funding

The study is supported by the Bangladesh Agricultural University Research System, 10.13039/100019278 (2022/12/BAU).

## Data Availability

The data that support the findings of this study are available from the corresponding author upon reasonable request.
